# Massachusetts Medical Society

**Published:** 1847-11

**Authors:** 


*Massachusetts Medical Society.*—A quarterly meeting of the Counsellors of this Society was held at the Masonic Temple on Wednesday last, the day the Journal is published, which precluded the possibility of inserting even a synopsis of the transactions in that No. There has not been so many Counsellors together at an ordinary business meeting within the compass of our recollection. The circumstance even quite astonished one gentleman, who expressed his surprise, and exclaimed—"Mr. President, what is the meaning of this unusual congregation? Is there some design in it?" Dr. Howe, of Billerica, contrary to an impression abroad, took the chair, having withdrawn his letter declining the honour of the presidency. A long and unnecessarily tedious debate occurred in regard to a short report from the delegates of the Society to the National Medical Convention. Closely upon that, another came upon the tapis, which would have been admirable in a legislative body, where the object was solely to occupy the time in order to keep off another subject.

Everything else having been satisfactorily disposed of, Dr. Childs, of Pittsfield, introduced the following resolution:—

"*Whereas*, The great object of medical association is the advancement of medical science, and the promotion of harmony and good feeling in the profession, thereby contributing to the best interests of society—and whereas the present organization of the Massachusetts Medical Society does not fully meet these important objects—therefore, *Resolved*, that a change in the organization of the Massachusetts Medical Society is in our opinion deemed both wise and expedient—and that the change consist in making the basis of the State Society, local or county associations; in other words, having the State Society constituted by delegates annually chosen by the county associations, agreeably to the principle adopted in the States of Connecticut, New York, Vermont, Ohio, and in most of the States in the Union."

Its introduction was like sending a fire-brand into a magazine of wet powder. There was no sudden explosion, but a general movement on the surface. By little and little, the ignition extended, and such a warming up of the quiescent old furnace has not occurred in that sedate circle for many a day. The Counsellor from Berkshire expressed himself with an unusual degree of energy and eloquence. Even those who most staunchly opposed the measures advocated by him for a re-organization of the Society, so that the profession in the western counties may profit by the association, admitted that the speaker was a man of strength, who pleaded his cause with commanding force and dignity.. We shall not detail the various propositions for throwing overboard the petition for a remodelling of this venerable institution, which has, for a period of sixty years, conducted so many physicians and surgeons in peace, security and respectability; nor advert to the cogent and ingenious arguments urged upon the Council in favour of the scheme. After a protracted, as well as excited session, an unmistaken evidence of impatience being manifested by those wishing to take the afternoon cars, as well as by another division accustomed to dining before tea-time, a large committee was raised, to whom the subject was referred, and a report may be expected at the next meeting in February, when, it may safely be predicted, the Council will again be well attended.

Drs. Jeffreys of Boston, Peirson of Salem, Walker of Boston, Bartlett of Concord, and Childs of Pittsfield, were the prominent speakers on this exciting question. While some regretted, in private conversation, the introduction of this apple of discord, others were delighted with the discovery that there was some excitability in what they had doubtless considered as dry bones.

When the Berkshire gentleman memorialized the General Court the past winter, and stated sundry grievances, such as the local wants of the members residing at a distance from Boston, and also the noticeable fact that about one hundred regularly-educated physicians, in western Massachusetts, could not become members of the Society in its present imperfect condition, the petition was very summarily put under the table, or, what was equivalent thereto, disregarded by the committee, which aroused the Æsculapian blood on the sun-setting side of the Berkshire hills, whose excited members now seem disposed, like General Taylor, never to surrender.—*Boston Med. and Surg. Journal.*

